# Recognizing Sequences of Sequences

**DOI:** 10.1371/journal.pcbi.1000464

**Published:** 2009-08-14

**Authors:** Stefan J. Kiebel, Katharina von Kriegstein, Jean Daunizeau, Karl J. Friston

**Affiliations:** 1 Wellcome Trust Centre for Neuroimaging, London, United Kingdom; 2 Max Planck Institute for Human Cognitive and Brain Sciences, Leipzig, Germany; Radboud University Nijmegen Medical Centre, Netherlands

## Abstract

The brain's decoding of fast sensory streams is currently impossible to emulate, even approximately, with artificial agents. For example, robust speech recognition is relatively easy for humans but exceptionally difficult for artificial speech recognition systems. In this paper, we propose that recognition can be simplified with an internal model of how sensory input is generated, when formulated in a Bayesian framework. We show that a plausible candidate for an internal or generative model is a hierarchy of ‘stable heteroclinic channels’. This model describes continuous dynamics in the environment as a hierarchy of sequences, where slower sequences cause faster sequences. Under this model, online recognition corresponds to the dynamic decoding of causal sequences, giving a representation of the environment with predictive power on several timescales. We illustrate the ensuing decoding or recognition scheme using synthetic sequences of syllables, where syllables are sequences of phonemes and phonemes are sequences of sound-wave modulations. By presenting anomalous stimuli, we find that the resulting recognition dynamics disclose inference at multiple time scales and are reminiscent of neuronal dynamics seen in the real brain.

## Author Summary

Despite tremendous advances in neuroscience, we cannot yet build machines that recognize the world as effortlessly as we do. One reason might be that there are computational approaches to recognition that have not yet been exploited. Here, we demonstrate that the ability to recognize temporal sequences might play an important part. We show that an artificial decoding device can extract natural speech sounds from sound waves if speech is generated as dynamic and transient sequences of sequences. In principle, this means that artificial recognition can be implemented robustly and online using dynamic systems theory and Bayesian inference.

## Introduction

Many aspects of our sensory environment can be described as dynamic sequences. For example, in the auditory domain, speech and music are sequences of sound-waves [1],[2], where speech can be described as a sequence of phonemes. Similarly, in the visual domain, speaking generates sequences of facial cues with biological motion [3],[4]. These auditory and visual sequences have an important characteristic: the transitions between the elements are continuous; i.e., it is often impossible to identify a temporal boundary between two consecutive elements. For example, phonemes (speech sounds) in a syllable are not discrete entities that follow each other like beads on a string but rather show graded transitions to the next phoneme. These transitions make artificial speech recognition notoriously difficult [5]. Similarly, in the visual domain, when we observe someone speaking, it is extremely difficult to determine exactly where the movements related to a phoneme start or finish. These dynamic sequences, with brief transitions periods between elements, are an inherent part of our environment, because sensory input is often generated by the fluent and continuous movements of other people, or indeed oneself.

Dynamic sequences are generated on various time-scales. For example, in speech, formants form phonemes and phonemes form syllables. Sequences, which exist at different time-scales, are often structured hierarchically, where sequence elements on one time-scale constrain the expression of sequences on a finer time-scale; e.g. a syllable comprises a specific sequence of phonemes. This functional hierarchy of time-scales may be reflected in the hierarchical, anatomical organisation of the brain [6]. For example, in avian brains, there is anatomical and functional evidence that birdsong is generated and perceived by a hierarchical system, where low levels represent transient acoustic details and high levels encode song structure at slower time-scales [7],[8]. An equivalent temporal hierarchy might also exist in the human brain for representing auditory information, such as speech [1], [9]–[12].

Here we ask the following question: How does the brain recognize the dynamic and ambiguous causes of noisy sensory input? Based on experimental and theoretical evidence [13]–[18] we assume the brain is a recognition system that uses an internal model of its environment. The structure of this model is critical: On one hand, the form of the model must capture the essential architecture of the process generating sensory data. On the other hand, it must also support robust inference. We propose that a candidate that fulfils both criteria is a model based on a hierarchy of stable heteroclinic channels (SHCs). SHCs have been introduced recently as a model of neuronal dynamics *per se* [19]. Here, we use SHCs as the basis of neuronal recognition, using an established Bayesian scheme for modelling perception [20]. This brings together two recent developments in computational approaches to perception: Namely, winnerless competition in stable heteroclinic channels and the hypothesis that the brain performs Bayesian inference. This is important because it connects a dynamic systems perspective on neuronal dynamics [19],[21],[22] with the large body of work on the brain as an inference machine [13]–[18].

To demonstrate this we generate artificial speech input (sequences of syllables) and describe a system that can recognize these syllables, online from incoming sound waves. We show that the resulting recognition dynamics display functional characteristics that are reminiscent of psychophysical and neuronal responses.

## Model

In this section, we describe an online recognition scheme for continuous sequences with hierarchical structure. This scheme rests on the concept of stable heteroclinic channels (SHCs) [23], which are combined with an online Bayesian inversion scheme [20]. We now describe these elements and how they are brought together. Note that all variables and their meaning are also listed in Table 1 and 2.

**Table 1:** Variables used for hierarchies of stable heteroclinic channels (SHCs).

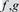	Nonlinear evolution and observation function
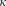	Scalar rate constant
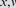	Hidden and causal state vectors
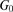 , 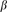 , 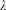	Scalar control parameters:
	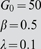
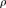	Inhibitory connectivity matrix
*S*	Sigmoid function
*w,z*	state and observation noise vectors
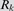	*k*th template connectivity matrix

This table lists all variables and their meaning for Eqs. 1 to 3. The additional superscript 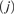 in Eqs. 2 and 3 denotes the level of the SHC, where level 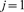 is the lowest.

**Table 2:** Variables used in Bayesian recognition scheme.

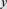	Sensory input vector
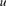	Concatenated hidden and causal state vectors 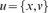
m	A model, which specifies the structure of likelihood and priors
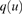	Recognition density used by recognition system to approximate the true but unknown generative density 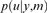
*F,U,S*	Free energy, energy, and entropy (scalars)
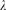	Sufficient statistics vector 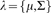 of normal recognition density *q*
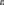	Prediction error vector (causal states)
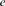	Prediction error vector (hidden states)

This table lists all variables used in Eqs. 4 to 8. Note that all variables except for *m* are functions of time.

### Stable heteroclinic channels (SHCs)

SHCs are attractors formed by artificial neuronal networks, which prescribe sequences of transient dynamics [22]–[25]. The key aspect of these dynamical systems is that their equations of motion describe a manifold with a series of saddle points. At each saddle point, trajectories are attracted from nearly all directions but are expelled in the direction of another saddle point. If the saddle points are linked up to form a chain, the neuronal state follows a trajectory that passes through all these points, thereby forming a sequence. These sequences are exhibited robustly, even in the presence of high levels of noise. In addition, the dynamics of the SHCs are itinerant due to dynamical instability in the equations of motion and noise on the states. This noise also induces a variation in the exact times that sequence elements are visited. This can be exploited during recognition, where the SHC places prior constraints on the sequence that elements (repelling fixed-points) are visited but does not constrain the exact timing of these visits.

The combination of these two features, robustness of sequence order but flexibility in sequence timing, makes the SHC a good candidate for the neuronal encoding of trajectories [19],[26]. Rabinovich et al. have used SHCs to explain how spatiotemporal neuronal dynamics observed in odour perception, or motor control of a marine mollusc, can be expressed in terms of a dynamic system [22],[27].

Varona et al. used Lotka-Volterra-type dynamics to model a network of six neurons in a marine mollusc [27]: With particular lateral inhibition between pairs of neurons and input to each neuron, the network displayed sequences of activity. Following a specific order, each neuron became active for a short time and became inactive again, while the next neuron became active, and so on. Stable heteroclinic channels rest on a particular form of attractor manifold that supports itinerant dynamics. This itinerancy can result from deterministic chaos in the absence of noise, which implies the presence of heteroclinic cycles. When noise is added, itinerancy can be assured, even if the original system has stable fixed-points. However, our motivation for considering stochastic differential equations is to construct a probabilistic model, where assumptions about the distribution of noise provide a formal generative model of sensory dynamics.

As reviewed in [22], Lotka-Volterra dynamics can be derived from simple neural mass models of mean membrane potential and mean firing rate [21]. Here, we use a different neural mass model, where the state-vector *x* can take positive or negative values:
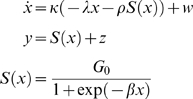
(1)where the motion of a hidden-state vector (e.g., mean membrane potentials) *x* is a nonlinear function of itself with scalar parameters 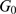, 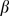, 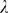 and a connectivity matrix 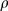. The hidden state-vector enters a nonlinear function *S* to generate outcomes (e.g., neuronal firing rates) *y*. Each element 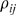 determines the strength of lateral inhibition from state *j* to *i*. Both the state and observation equations above include additive normally distributed noise vectors *w* and *z*. When choosing specific parameter values (see below), the states display stereotyped sequences of activity [28]. Rabinovich et al. [19] termed these dynamics ‘stable heteroclinic channels’ (SHCs). If the channel forms a ring, once a state is attracted to a saddle point, it will remain in the SHC.

SHCs represent a form of itinerant dynamics [26],[29],[30] and may represent a substrate for neuronal computations [31]. Remarkably, the formation of SHCs seems to depend largely on the lateral inhibition matrix 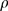 and not on the type of neuronal model; see Ivanchenko et al. [32] for an example using a complex two-compartment spiking neuron model.

In this paper, we propose to use SHCs not as a model for neuronal dynamics *per se* but as a generative model of how sensory input is generated. This means that we interpret *x* as hidden states in the environment, which generate sensory input *y*. The neuronal response to sampling sensory input *y* are described by recognition dynamics, which decode or deconvolve the causes *x* from that input. These recognition dynamics are described below. This re-interpretation of Eq. 1 is easy to motivate: sensory input is usually generated by our own body and other organisms. This means input is often generated by neuronal dynamics of the sort described in Eq. 1.

### Hierarchies of stable heteroclinic channels

A SHC can generate repetitive, stereotyped sequences. For example, in a system with four saddle points, an SHC forces trajectories through the saddle points in a sequence, e.g. ‘1-2-3-4-1-2-3-4-1…’. In contrast, a SHC cannot generate ‘1-2-3-4-3-4-2-1…’, because the sequence is not repetitive. However, to model sensory input, for example speech, one must be able to recombine basic sequence-elements like phonemes in ever-changing sequences. One solution would be to represent each possible sequence of phonemes (e.g. each syllable) with a specific SHC. A more plausible and parsimonious solution is to construct a hierarchy of SHCs, which can encode sequences generated by SHCs whose attractor topology (e.g. the channels linking the saddle points) is changed by a supraordinate SHC. This can be achieved by making the connectivity matrix 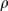 at a subordinate level a function of the output states of the supra-ordinate level. This enables the hierarchy to generate sequences of sequences to any hierarchical depth required.

Following a recent account of how macroscopic cortical anatomy might relate to time-scales in our environment [6], we can construct a hierarchy by setting the rate constant 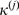 of the *j*-th level to a rate that is slower than its subordinate level, 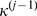. As a result, the states of subordinate levels change faster than the states of the level above. This means the control parameters 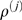 at any level change more slowly than its states, 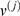; because the slow change in the attractor manifold is controlled by the supraordinate states:
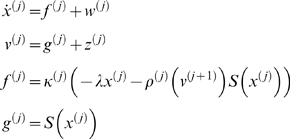
(2)where the superscript indexes level *j* (level 1 being the lowest level), 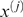 are ‘hidden states’, and 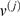 are outputs to the subordinate level, which we will call ‘causal states’. As before, at the first level, 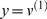 is the sensory stream. In this paper, we consider hierarchies with relative time-scales 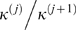 of around four. This means that the time spent in the vicinity of a saddle point at a supraordinate level is long enough for the subordinate level to go through several saddle points. As before, all levels are subject to noise on the motion of the hidden states 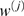 and the causal states 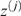. At the highest level, the control parameters, 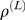 are constant over time. At all other levels, the causal states of the supraordinate level, 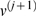, enter the subordinate level by changing the control parameters, the connectivity matrix 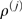:
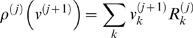
(3)Here, 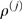 is a linear mixture of ‘template’ control matrices 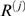, weighted by the causal states at level 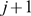. Each of these templates is chosen to generate a SHC. Below, we will show examples of how these templates can be constructed to generate various sequential phenomena. The key point about this construction is that states from the supraordinate level select which template controls the dynamics of the lower level. By induction, the states at each level follow a SHC because the states at the supraordinate level follow a SHC. This means only one state is active at any time and only one template is selected for the lower level. An exception to this is the transition from one state to another, which leads to a transient superposition of two SHC-inducing templates (see below). Effectively, the transition transient at a specific level gives rise to brief spells of non-SHC dynamics at the subordinate levels (see results). These transition periods are characterized by dissipative dynamics, due to the largely inhibitory connectivity matrices, inhibition controlled by parameter 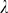 (Eq. 2) and the saturating nonlinearity *S*.

In summary, a hierarchy of SHCs generates the sensory stream 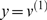 at the lowest (fastest) level, which forms a sequence of sequences expressed in terms of first-level states. In these models, the lower level follows a SHC, i.e. the states follow an itinerant trajectory through a sequence of saddle points. This SHC will change whenever the supraordinate level, which follows itself a SHC, moves from one saddle point to another. Effectively, we have constructed a system that can generate a stable pattern of transients like an oscillator; however, as shown below, the pattern can have deep or hierarchical structure. Next, we describe how the causes 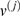 can be recognized or deconvolved from sensory input *y*.

### Bayesian recognition using SHC hierarchies and the free-energy principle

We have described how SHCs can, in principle, generate sequences of sequences that, we assume, are observed by an agent as its input *y*. To recognise the causes of the sensory stream the agent must infer the hidden states online, i.e. the system does not look into the future but recognizes the current states 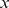 and 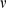 of the environment, at all levels of the hierarchy, by the fusion of current sensory input and internal dynamics elicited by past input. An online recognition scheme can be derived from the ‘free-energy principle’, which states that an agent will minimize its surprise about its sensory input, under a model it entertains about the environment; or, equivalently maximise the evidence for that model [18]. This requires the agent to have a dynamic model, which relates environmental states to sensory input. In this context, recognition is the Bayesian inversion of a generative model. This inversion corresponds to mapping sensory input to the posterior or conditional distribution of hidden states. In general, Bayesian accounts of perception rest on a generative model. Given such a model, one can use the ensuing recognition schemes in artificial perception and furthermore compare simulated recognition dynamics (in response to sensory input), with evoked responses in the brain. The generative model in this paper is dynamical and based on the nonlinear equations 1 and 2. More precisely, these stochastic differential equations play the role of empirical priors on the dynamics of hidden states causing sensory data.

In the following, we review briefly, the Bayesian model inversion described in [20] for stochastic, hierarchical systems and apply it, in the next section, to hierarchical SHCs.

Given some sensory data vector *y*, the general inference problem is to compute the model evidence or marginal likelihood of y, given a model *m*:
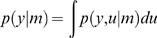
(4)where the generative model 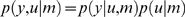 is defined in terms of a likelihood 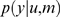 and prior 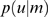 on hidden states. In Equation 4, the state vector 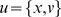 subsumes the hidden and causal states at all levels of a hierarchy (Eq. 2). The model evidence can be estimated by converting this difficult integration problem (Eq. 4) into an easier optimization problem by optimising a free-energy bound on the log-evidence [33]. This bound is constructed using Jensen's inequality and is a function of an arbitrary *recognition* density, 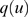:
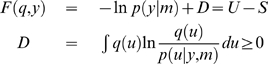
(5)The free-energy comprises an energy term 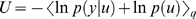 and an entropy term 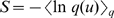 and is defined uniquely, given a generative model 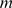. The free-energy is an upper bound on the surprise or negative log-evidence, because the Kullback-Leibler divergence 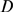, between the recognition and conditional density, is always positive. Minimising the free-energy minimises the divergence, rendering the recognition density 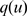 an approximate conditional density. When using this approach, one usually employs a parameterized fixed-form recognition density, 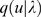 [20]. Inference corresponds to optimising the free-energy with respect to the sufficient statistics, 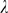 of the recognition density:
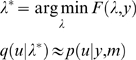
(6)The optimal statistics 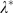 are sufficient to describe the approximate posterior density; i.e. the agent's belief about (or representation of) the trajectory of the hidden and causal states. We refer the interested reader to Friston et al. [34] for technical details about this variational Bayesian treatment of dynamical systems. Intuitively, this scheme can be thought of as augmented gradient descent on a free-energy bound on the model's log-evidence. Critically, it outperforms conventional Bayesian filtering (e.g., Extended Kalman filtering) and eschews the computation of probability transition matrices. This means it can be implemented in a simple and neuronally plausible fashion [20].

In short, this recognition scheme operates online and recognizes current states of the environment by combining current sensory input with internal recognition dynamics, elicited by past input.

A recognition system that minimizes its free-energy efficiently will come to represent the environmental dynamics in terms of the sufficient statistics of recognition density; e.g. the conditional expectations and variances of 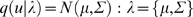. We assume that the conditional moments are encoded by neuronal activity; i.e., Equation 6 prescribes neuronal recognition dynamics. These dynamics implement Bayesian inversion of the generative model, under the approximations entailed by the form of the recognition density. Neuronally, Equation 6 can be implemented using a message passing scheme, which, in the context of hierarchical models, involves passing prediction errors up and passing predictions down, from one level to the next. These prediction errors are the difference between the causal states (Equation 2);
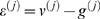
(7)at any level *j*, and their prediction from the level above, evaluated at the conditional expectations [18],[35]. In addition, there are prediction errors that mediate dynamical priors on the motion of hidden states within each level (Equation 2);
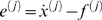
(8)This means that neuronal populations encode two types of dynamics: the conditional expectations of states of the world and the prediction errors. The dynamics of the first are given by Equation 6, which can be formulated as a function of prediction error. These dynamics effectively suppress or explain away prediction error; see [34] for details.

This inversion scheme is a generic recognition process that receives dynamic sensory input and can, given an appropriate generative model, rapidly identify and track environmental states that are generating current input. More precisely, the recognition dynamics resemble the environmental (hidden) states they track (to which they are indirectly coupled), but differ from the latter because they are driven by a gradient descent on free-energy; Eq. 6 (i.e. minimize prediction errors: Eqs. 7 and 8). This is important, because we want to use SHCs as a generative model, not as a model of neuronal encoding *per se*. This means that the neuronal dynamics will only recapitulate the dynamics entitled by SHCs in the environment, if the recognition scheme can suppress prediction errors efficiently in the face of sensory noise and potential beliefs about the world.

We are now in a position to formulate hierarchies of SHCs as generative models, use them to generate sensory input and simulate recognition of the causal states generating that input. In terms of low-level speech processing, this means that any given phoneme will predict the next phoneme. At the same time, as phonemes are recognized, there is also a prediction about which syllable is the most likely context for generating these phonemes. This prediction arises due to the learnt regularities in speech. In turn, the most likely syllable predicts the next phoneme. This means that speech recognition can be described as a dynamic process, on multiple time-scales, with recurrently evolving representations and predictions, all driven by the sensory input.

### A model of speech recognition

In the auditory system, higher cortical levels appear to represent features that are expressed at slower temporal scales [36]. Wang et al. [37] present evidence from single-neuron recordings that there is a ‘slowing down’ of representational trajectories from human auditory sensory thalamus (a ‘relay’ to the primary auditory cortex), the medial geniculate body (MGB) to primary auditory cortex (AI). In humans, it has been found that the sensory thalamus responds preferentially to faster temporal modulations of sensory signals, whereas primary cortex prefers slower modulations [10]. These findings indicate that neuronal populations, at lower levels of the auditory system (e.g. MGB), represent faster environmental trajectories than higher levels (e.g., A1). Specifically, the,MGB responds preferentially to temporal modulations of ∼20 Hz (∼50 ms), whereas AI prefers modulations at ∼6 Hz (∼150 ms) [10]. Such a temporal hierarchy would be optimal for speech recognition, in which information over longer time-scales provides predictions for processing at shorter time scales. In accord with this conjecture, optimal encoding of fast (rapidly modulated) dynamics by top-down predictions has been found to be critical for communication [1],[12],[38].

**Figure 1:**
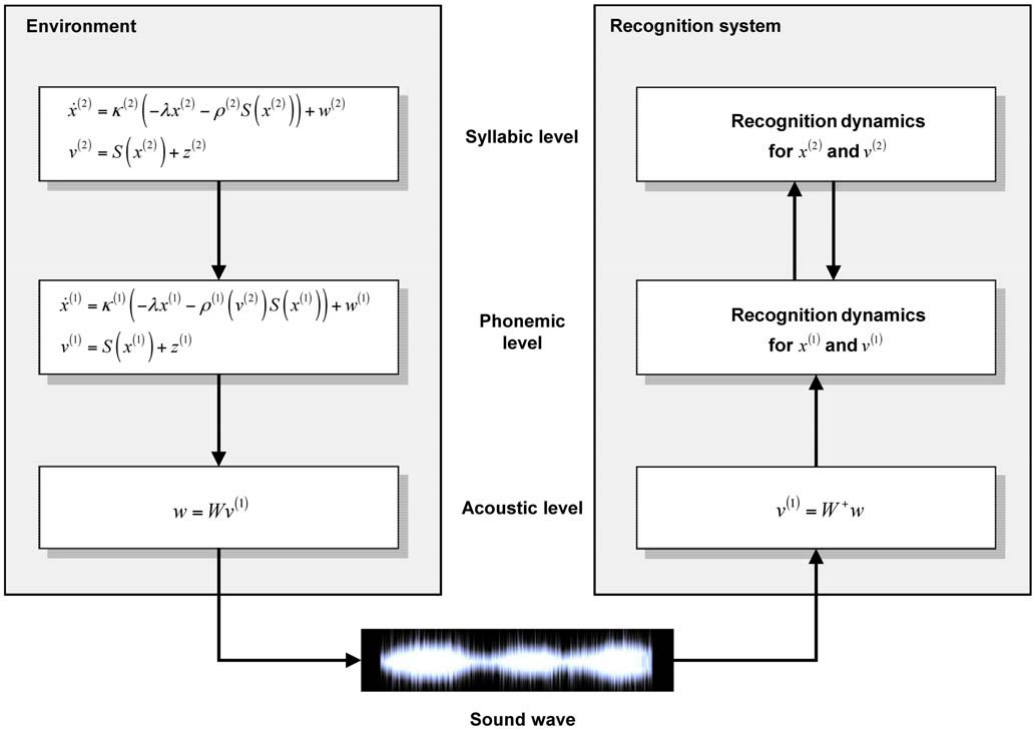
Schematic of the generative model and recognition system. This schematic shows the equations which define both the generation of stimuli (left, see Equation 2) and the recognition scheme based on a generative model. There are three levels; the phonemic and syllabic levels employ stable heteroclinic channels, while the acoustic level is implemented by a linear transform. 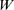 corresponds to sound file extracts and 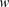 is the resulting sound wave. This sound wave is input to the recognition system, with a linear (forward) projection using the pseudo-inverse 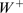. The recognition of the phonemic and syllabic level uses bottom-up and top-down message passing between the phonemic and syllabic level, following Equation 6.

We model this ‘slowing down’ with a hierarchical generative model based on SHCs. This model generates sequences of syllables, where each syllable is a sequence of phonemes. Phonemes are the smallest speech sounds that distinguishes meaning and a syllable is a unit of organization for a sequence of phonemes. Each phoneme prescribes a sequence of sound-wave modulations which correspond to sensory data. We generated data in this fashion and simulated online recognition (see Figure 1). By recognizing speech-like phoneme-sequences, we provide a proof-of-principle that a hierarchical system can use sensory streams to infer sequences. This not only models the slowing down of representations in the auditory system [10],[12],[37],[38], but may point to computational approaches to speech recognition. In summary, the recognition dynamics following Equation 6 are coupled to a generative model based on SHCs via sensory input. The systems generating and recognising states in Fig. 1 are both dynamic systems, where a non-autonomous recognition system is coupled to an autonomous system generating speech.

**Figure 2:**
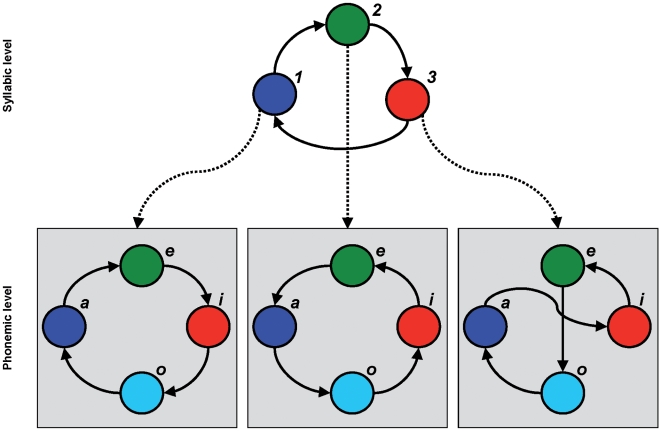
Two-level model to generate phoneme sequences. Schematic illustration of the phoneme sequence generation process. At the syllabic level, one of three syllables is active and induces a specific lateral connectivity structure at the phonemic level. The transition speed at the phonemic level is four times faster than at the syllabic level. The resulting phoneme and syllable dynamics of the model are shown in Fig. 3a.

All our simulations used hierarchies with two levels (Figure 2). The first (phonemic) level produces a sequence of phonemes, and the second (syllabic) level encodes sequences of syllables. We used Equation 2 to produce phoneme sequences, where the generating parameters are listed in Table 3. The template matrices 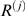 (Equation 3) were produced in the following way: We first specified the sequence each template should induce; e.g., sequence 1-2-3 for three neuronal populations. We then set elements on the main diagonal to 1, the elements (2,1), (3,2), (1,3) to value 0.5, and all other elements to 5 [28]. More generally for sequence 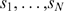


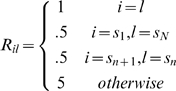
(9)Note that SHC hierarchies can be used to create a variety of different behaviours, using different connectivity matrices. Here we explore only a subset of possible sequential dynamics.

**Table 3:** Default parameters used for simulations with Equations 2 and 3.

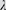	0.3
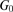	50
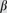	0.5
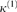	1/8
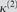	1/32

When generating sensory data *y*, we added noise 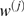 and 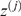 to both the hidden and causal states. At the first and second levels, this was normally distributed zero-mean noise with log-precisions of ten and sixteen, respectively. These noise levels were chosen to introduce noisy dynamics but not to the extent that the recognition became difficult to visualise. We repeated all the simulations reported below with higher noise levels and found that the findings remained qualitatively the same (results not shown). Synthetic stimuli were generated by taking a linear mixture of sound waves extracted from sound files, in which a single speaker pronounced each of four vowel-phonemes: [a], [e], [i], [o]. These extracts *W* were sampled at 22050 Hz and about 14 ms long. The mixture was weighted by the causal states of the phonemic level; 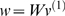. This resulted in a concatenated sound wave file *w*. When this sound file is played, one perceives a sequence of vowels with smooth, overlapping transitions (audio file S1). These transitions are driven by the SHCs guiding the expression of the phonemes and syllables at both levels of the generative hierarchy.

For computational simplicity, we circumvented a detailed generative model of the acoustic level. For simulated recognition, the acoustic input (the sound wave) was transformed to phonemic input by inverting the linear mixing described above every seven ms of simulated time (one time bin). This means that our recognition scheme at the acoustic level assumes forward processing only (Fig. 1). However, in principle, given an appropriate generative model [39],[40], one could invert a full acoustic model, using forward and backward message passing between the acoustic and phonemic levels.

## Results

In this section, we illustrate that the recognition scheme described above can reliably decode syllabic and phonemic structure from sensory input online, if it has the correct generative model. We will also describe how recognition fails when the generative model does not have a form that provides veridical predictions of the sensorium, e.g., when agents are not conspecific or we hear a foreign language. These simulations relate to empirical studies of brain responses evoked by unpredicted linguistic stimuli. We conclude with a more subtle violation that we deal with in everyday audition; namely the recognition of speech presented at different speeds.

### Recognising a sequence of sequences

**Figure 3:**
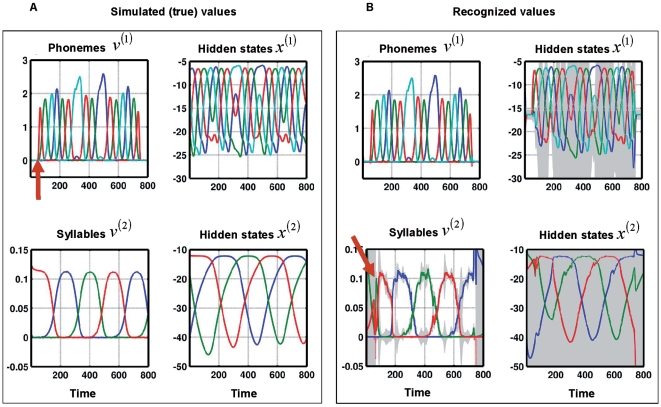
Recognition of a sequence of sequences. (A): Dynamics of generated causal and hidden states at the phonemic and syllabic level, using Equation 2. At the syllabic level, there are three different syllables (1: blue, 2: green, 3: red), following the syllable sequence 1→2→3. The slowly changing state Syllable 1 causes the faster-moving phoneme sequence a→e→i→o (blue→green→red→cyan), syllable 2: o→i→e→a (cyan→red→green→blue), and syllable 3: a→i→e→o (blue→red→green→cyan). See Fig 2 for a schematic description of these sequences. At the beginning and end of the time-series 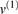 (top-left plot), we introduced silence by applying a windowing function to zero time points 0 to 50 and 750 to 800. The red arrow indicates the end of the initial silent period. The phonemic states 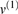 cause sound waves, resolved at 22050 Hz (see Fig. 1). These sound waves are the input to the recognition system. (B): The recognition dynamics after inverting the sound wave. At the phonemic level, the states follow the true states closely. At the syllabic level, the recognized causal state dynamics 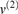 are rougher than the true states but track the true syllable sequence veridically. The high-amplitude transients of 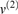 at the beginning and end of the time-series are due to the silent periods, where the syllabic recognition states 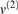 experience high uncertainty (plotted in grey: confidence intervals of 95% around the mean). Note that the hidden states, at both levels, experience high uncertainty whenever a phoneme or syllable is inactive. The red arrow indicates an initial but rapidly corrected mis-recognition of the causing syllable.

To create synthetic stimuli we generated syllable sequences consisting of four phonemes or states; [a], [e], [i], and [o], over 11.25 seconds (800 time points), using a two-level SHC model (Fig. 2). To simulate word-like stimuli, we imposed silence at the beginning and the end by windowing the phoneme sequence (Fig. 3A, top left). At the syllabic level, we used three syllables or states to form the second-level sequence (1–2–3)^(2)^; where the numbers denote the sequence and the superscript indicates the sequence level. The three causal states 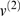 of the syllabic level entered the phonemic level as control parameters to induce their template matrices as in Equation 3. This means that each of the three syllable states at the second level causes a phoneme sequence at the first: 
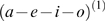
, 
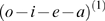
, and 
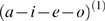
, see Fig. 2 and listen to the audio file S1. In Fig. 3A we show the causal and hidden states, at both levels, generated by this model. The remaining parameters, for both levels, are listed in Table 3. Note that the rate constant of the syllabic level is four times slower than at the phonemic level. As expected, the phoneme sequence at the first level changes as a function of the active syllable at the second level. The transients caused by transitions between syllables manifest at the first level as temporary changes in the amplitude or duration of the active phoneme.

We then simulated recognition of these sequences. Fig. 3B shows that our recognition model successfully tracks the true states at both levels. Note the recognition dynamics rapidly ‘lock onto’ the causal states from the onset of the first phoneme of the first syllable (time point 50). Interestingly, the system did not recognize the first syllable (true: syllable 3 (red line), recognized: syllable 2 (green line) between time points 50 to 80 (see red arrow in Fig. 3B), but corrected itself fairly quickly, when the sensory stream indicated a new phoneme that could only be explained by the third syllable. This initial transient at the syllabic level shows that recognition dynamics can show small but revealing deviations from the true state dynamics. In principle, these deviations could be used to test whether the real auditory system uses a recognition algorithm similar to the one proposed; in particular, the simulated recognition dynamics could be used to explain empirical neurophysiological responses.

### Sensitivity to sequence violations

What happens if the stimuli deviate from learned expectations (e.g. violation of phonotactic rules)? In other words, what happens if we presented known phonemes that form unknown syllables? This question is interesting for two reasons. First, our artificial recognition scheme should do what we expect real brains to do when listening to a foreign language: they should be able to recognize the phonemes but should not derive high-order ‘meaning’ from them; i.e. should not recognize any syllable. Secondly, there are well-characterised brain responses to phonotactic violations, e.g. [41]–[43]. These are usually event-related responses that contain specific waveform components late in peristimulus time, such as the N400. The N400 is an event-related potential (ERP) component typically elicited by unexpected linguistic stimuli. It is characterized as a negative deflection (topologically distributed over central-parietal sites on the scalp), peaking approximately 400 ms after the presentation of an unexpected stimulus.

**Figure 4:**
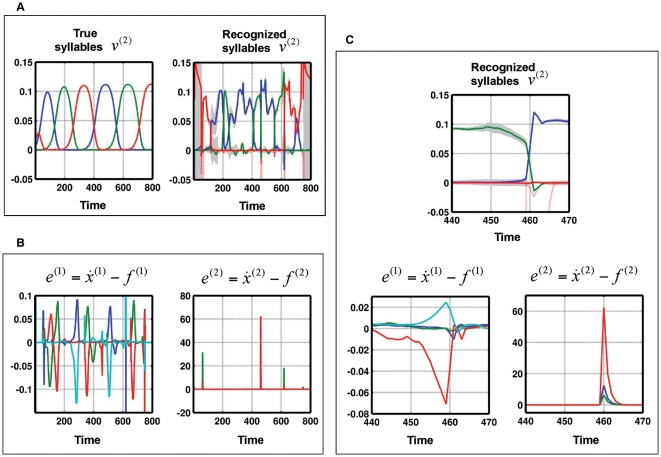
Recognition of sequences with phonotactic violation. (A): True and recognized syllable dynamics of a two-level model when the syllables are unknown to the recognition system. Left: True dynamics of 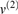, Right: Recognition dynamics for 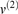. (B): Left: Prediction error 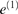 at phonemic level. Right: Prediction error 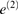 at syllabic level. (C): Zoom of dynamics shown in A and B from time points 440 to 470. See text for description of these dynamics.

To model phonotactic violations, we generated data with the two-level model presented above. However, we used syllables, i.e. sequences of phonemes, that the recognition scheme was not informed about and consequently could not recognise (it has three syllables in its repertoire: 
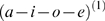
, 
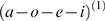
, and 
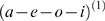
). Thus the recognition scheme knows all four phonemes but is unable to predict the sequences heard. Fig. 4A shows that the recognition system cannot track the syllables; the recognized syllables are very different from the true syllable dynamics. At the phonemic level, the prediction error 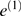 deviates from zero whenever a new (unexpected) phoneme is encountered (Fig. 4B). The prediction error at the syllabic level is sometimes spike-like and can reach high amplitudes, relative to the typical amplitudes of the true states (see Fig. 4A and B). This means that the prediction error signals violation of phonotactic rules. In Fig. 4C, we zoom in onto time points 440 to 470 to show how the prediction error evolves when evidence of a phonotactic violation emerges: At the phoneme level, prediction error builds up because an unexpected phoneme appears. After time point 450, the prediction error 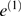 grows quickly, up to the point that the system resolves the prediction error. This is done by ‘switching’ to a new syllable, which can explain the transition to the emerging phoneme. The switching creates a large amplitude prediction error 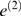 at time point 460. In other words, in face of emerging evidence that its current representation of syllables and phonemes cannot explain sensory input, the system switches rapidly to a new syllable representation, giving rise to a new prediction error. It may be that these prediction errors are related to electrophysiological responses to violations of phonotactic rules, [44],[45]. This is because the largest contributors to non-invasive electromagnetic signals are thought to be superficial pyramidal cells. In biological implementations of the recognition scheme used here [20], these cells encode prediction error.

In summary, these simulations show that a recognition system cannot represent trajectories or sequences that are not part of its generative model. In these circumstances, recognition experiences intermittent high-amplitude prediction errors because the internal predictions do not match the sensory input. There is a clear formal analogy between the expression of prediction error in these simulations and mismatch or prediction violation responses observed empirically. The literature that examines event-related brain potentials (ERPs) and novelty processing “reveals that the orienting response engendered by deviant or unexpected events consists of a characteristic ERP pattern, comprised sequentially of the mismatch negativity (MMN) and the novelty P3 or P3a” [46].

### Robustness to speed of speech

Human speech recognition is robust to the speed of speech [47],[48]. How do our brains recognize speech at different rates? There are two possible mechanisms in our model that can deal with ‘speaker speed’ parameters online. First, one could make the rate constants 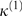 and 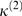 free parameters and optimise them during inversion. Adjusting to different speaker parameters is probably an essential faculty, because people speak at different speeds [49]. The second mechanism is that the recognition itself might be robust to deviations from the expected rate of phonemic transitions; i.e., even though the recognition uses the rate parameters appropriate for much slower speech, it still can recognize fast speech. This might explain why human listeners can understand speech at rates that they have never experienced previously [47]. In the following, we show that our scheme has this robustness.

**Figure 5:**
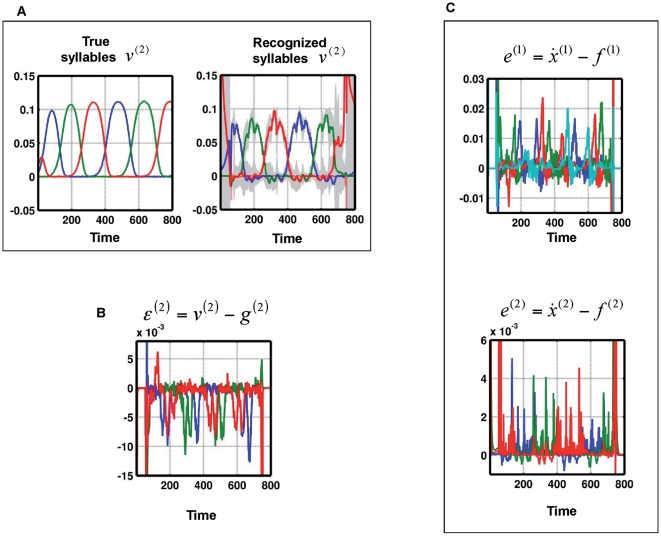
Recognition of unexpectedly fast phoneme sequences. (A): True and recognized syllable dynamics of a two-level model when the phoneme sequence is generated with a rate constant of 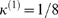 but recognized with a rate constant of 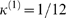, i.e. speech was 50% faster than expected. Left: True dynamics of 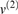, Right: Recognition dynamics for 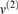. (B): Prediction error 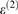 at syllabic level. (C) Top: Prediction error 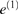 at phonemic level. Bottom: Prediction error 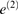 at syllabic level.

To simulate speed differences we used the same two-level model as in the simulations above with 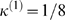 for the generation of phonemes, but with 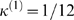 for recognition so that the stimulus stream was 50% faster than expected. As can be seen in Fig. 5A, the recognition can successfully track the syllables. This was because the second level supported the adaption to the fast sensory input by changing its recognition dynamics in responses to prediction error (see Fig. 5B: note the amplitude difference in Fig. 5A between the true and recognized 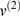). The prediction errors at both levels, 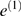 and 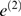, are shown in Fig. 5C. In particular, the second-level error 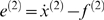 displayed spike-like corrections around second-level transitions. These are small in amplitude compared to both the amplitude of the hidden states and the prediction errors of the previous simulation (Fig. 4B). These results show that the system can track the true syllables veridically, where the prediction error accommodates the effects caused by speed differences. This robustness to variations in the speed of phoneme transitions might be a feature shared with the auditory system [50].

## Discussion

We have shown that stable heteroclinic channels (SHCs) can be used as generative models for online recognition. In particular, we have provided proof-of-concept that sensory input generated by these hierarchies can be deconvolved to disclose the hidden states causing that input. This is a non-trivial observation because nonlinear, hierarchical and stochastic dynamical systems are difficult to invert online [51],[52]. However, we found that the inversion of models based on SHCs is relatively simple. Furthermore, the implicit recognition scheme appears robust to noise and deviations from true parameters. This suggests that SHCs may be a candidate for neuronal models that contend with the same problem of de-convolving causes from sensory consequences. Moreover, hierarchical SHCs seem, in principle, an appropriate description of natural sequential input, which is usually generated by our own body or other organisms, and can be described as a mixture of transients and discrete events.

The general picture of recognition that emerges is as follows: Sensory input is generated by a hierarchy of dynamic systems in the environment. We couple this dynamic system, via sensory sampling, to our recognition system implementing the inversion dynamics (Fig. 1). The recognition system minimizes a proxy for surprise or model evidence; the negative free-energy (Eq. 6). To do this, the states of the recognition system move on manifolds, defined through the free-energy by the generative model. Here, we use a hierarchy of SHCs as generative model so that the manifold changes continuously at various time-scales. The inferred SHC states never reach a fixed point, but are perpetually following a trajectory through state-space, in the attempt to mirror the generative dynamics of the environment. When sensory input is unexpected (see second simulation, Fig. 4), the system uses the prediction error to change its representation quickly, at all levels, such that it best explains the sensory stream.

In a previous paper [6], we have shown that one can use chaotic attractors (i.e., a hierarchy of Lorenz attractors) to model auditory perception. However, SHCs may provide a more plausible model of sensory dynamics: First, they show structure over extended temporal scales, much like real sensory streams. This may reflect the fact that the processes generating sensory data are themselves (usually) neuronal dynamics showing winnerless competition. Secondly, many chaotic systems like the Lorenz attractor have only few states and cannot be extended to high dimensions in a straightforward fashion. This was no problem in our previous model, where we modelled a series of simple chirps, with varying amplitude and frequency [6]. However, it would be difficult to generate sequences of distinct states that populate a high dimensional state-space; e.g. phonemes in speech. In contrast, stable heteroclinic channels can be formulated easily in high dimensional state spaces.

In this paper, we used a generative model which was formally identical to the process actually generating sensory input. We did this for simplicity; however, any generative model that could predict sensory input would be sufficient. In one sense, there is no true model because it is impossible to disambiguate between models that have different forms but make the same predictions. This is a common issue in ill-posed inverse problems, where there are an infinite number of models that could explain the same data. In this context the best model is usually identified as the most parsimonious. Furthermore, we are not suggesting that all aspects of perception can be framed in terms of the inversion of SHCs; we only consider recognition of those sensory data that are generated by mechanisms that are formally similar to the itinerant but structured dynamics of SHCs.

The proof-of-concept presented above makes the SHC hierarchy a potential candidate for speech recognition models. The recognition dynamics we simulated can outpace the dynamics they are trying to recognise. In all our simulations, after some initial transient, the recognition started tracking the veridical states early in the sequence. For example, the scheme can identify the correct syllable before all of its phonemes have been heard. We only simulated two levels, but this feature of fast recognition on exposure to brief parts of the sequence may hold for many more levels. Such rapid recognition of potentially long sequences is seen in real systems; e.g., we can infer that someone is making a cup of tea from observing a particular movement, like getting a teabag out of a kitchen cupboard. The reason why recognition can be fast is that the generative model is nonlinear (through the top-down control of attractor manifolds). With nonlinearities, slow time-scales in hierarchical sequences can be recognized rapidly because they disclose themselves in short unique sequences in the sensory input. Furthermore, we demonstrated another requirement for efficient communication: recognition signals, via prediction error, when unrecognised syllables cannot be decoded with its phonotactic model. This is important, because, an agent can decide online whether its decoding of the message is successful or not. Following the free-energy principle, this would oblige the agent to act on its environment, so that future prediction error is minimized [18]. For example, the prediction error could prompt an action (‘repeat, please’) and initiate learning of new phonotactic rules.

Another aspect of SHC-based models is that they can recombine sensory primitives like phonemes in a large number of ways. This means that neuronal networks implementing SHC dynamics, based on a few primitives at the first level, can encode a large number of sequences. This feature is critical for encoding words in a language; e.g., every language contains many more words than phonemes [53]. The number of sequences that a SHC system can encode is
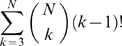
(10)where *N* is the number of elements [22]. This would mean, in theory, that the number of states that can be encoded with a sequence, given a few dozens primitives, is nearly endless. It is unlikely that this full capacity is exploited in communication. Rather, for efficient communication, it might be useful to restrict the number of admissible sequences to make them identifiable early in the sequence.

We did not equip the recognition model with a model of the silent periods at the beginning and end of a word (Fig. 3A). It is interesting to see how recognition resolves this: to approximate silence, the system held hidden phoneme states very negative by driving the states away from the SHC attractor and tolerating the violation of top-down predictions. However, the tolerance is limited as can be seen by the slightly positive inferred hidden states (Fig. 3B). Such behaviour is beneficial for recognition because the agent, within bounds, can deviate from internal predictions. A built-in error tolerance which is sensitive to the kind of errors it should endure to make recognition robust is important in an uncertain world. Robustness to errors would be impossible with an inversion scheme based on a deterministic model, which assumes that the sensory input follows a deterministic trajectory without any noise on the environmental causes. With such a recognition system, the agent could not deal with (unexpected) silence, because the SHC-based inversion dynamics would attract the state-trajectory without any means of resolving the resulting prediction error between the zero (silent) sensory input and the internal predictions. Recognition schemes based on stochastic systems can deviate adaptively from prior predictions, with a tolerance related to the variance of the stochastic innovations. Optimising this second-order parameter then becomes critical for recognition (see [20]).

### Links to neuroscience

There is emerging evidence in several areas of neuroscience that temporal hierarchies play a critical role in brain function [6]. The three areas where this is most evident are auditory processing [12], [37], [54]–[56], cognitive control [57]–[59], and motor control [60]. Our conclusions are based on a generic recognition scheme [20] and are therefore a consequence of our specific generative model, a temporal hierarchy of SHCs. This hierarchy of time-scales agrees well with the temporal anatomy of the hierarchical auditory system, where populations close to the periphery encode the fast acoustics, while higher areas form slower representations [9],[10],[37],[38],[61],[62]. In particular, our model is consistent with findings that phonological (high) levels have strong expectations about the relevance of acoustic (low) dynamics [38].

Neurobiological treatments of the present framework suppose that superficial pyramidal cell populations encode prediction error; it is these cells that contribute most to evoked responses as observed in magneto/electroencephalography (M/EEG) [63]. There is an analogy between the expression of prediction error in our simulations and mismatch or prediction violation responses observed empirically. In our simulations, prediction error due to a deviation from expectations is resolved by all levels (Fig. 4B). This might be an explanation for prominent responses to prediction violations to be spatially distributed, e.g., the mismatch negativity, the P300, and the N400 all seem to involve various brain sources in temporal and frontal regions [45], [46], [64]–[66]. Inference on predictable auditory streams has been studied and modelled in several ways, in an attempt to explain the rapid recognition of words in the context of sentences, e.g., [38], [67]–[70]. Our simulations show how, in principle, these accounts might be implemented in terms of neuronal population dynamics.

### Links to computational models

Learning, storing, inferring and executing sequences is a key topic in experimental [71]–[79], and theoretical neurosciences [80]–[82]; and robotics [83]–[86]. An early approach to modelling sequence processing focussed on feed-forward architectures. However, it was realised quickly that these networks could not store long sequences, because new input overwrote the internal representation of past states. The solution was to introduce explicit memory into recurrent networks, in various forms; e.g. as contextual nodes or ‘short-term memory’ [87],[88]. Although framed in different terms, these approaches can be seen as an approximation to temporal hierarchies, where different units encode representations at different time-scales.

A central issue in modelling perception is how sequences are not just recalled but used as predictions for incoming sensory input. This requires the ‘dynamic fusion’ of bottom-up sensory input and top-down predictions, Several authors e.g., [83], [89]–[92] use recurrent networks to implement this fusion. Exact Bayesian schemes based on discrete hierarchical hidden Markov models, specified as a temporal hierarchy, have been used to implement memory and recognition [93]. Here, we have used the free-energy principle (i.e. variational Bayesian inference on continuous hierarchical dynamical systems) to show how the ensuing recognition process leads naturally to a scheme which can deal with fast sequential inputs.

In conclusion, we have described a scheme for inferring the causes of sensory sequences with hierarchical structure. The key features of this scheme are: (i) the ability to describe natural sensory input as hierarchical and dynamic sequences, (ii) modeling this input using generative models, (iii) using dynamic systems theory to create plausible models, and (iv) online Bayesian inversion of the resulting models. This scheme is theoretically principled but is also accountable to the empirical evidence available from the auditory system; furthermore, the ensuing recognition dynamics are reminiscent of real brain responses.

## Supporting Information

Audio S1 Phoneme sequence generated in first simulation - mpg-file containing phoneme sequence sampled at 22050 Hz. The time courses of the four vowels can be seen in Fig. 3A (top left).
(0.18 MB MPG)
